# Sociodemographic factors in the development of Burnout Syndrome in South and Central American physicians

**DOI:** 10.15446/rsap.V25n2.105798

**Published:** 2023-03-01

**Authors:** Sandy N. Vásquez-Navas, Enrique Gea-Izquierdo

**Affiliations:** 1 SV: MD. Facultad de Medicina, Pontificia Universidad Católica del Ecuador. Quito, Ecuador. svasquez994@puce.edu.ec Pontificia Universidad Católica del Ecuador Facultad de Medicina Pontificia Universidad Católica del Ecuador Quito Ecuador svasquez994@puce.edu.ec; 2 EG: Biol. Ph.D. Medicina. Pontificia Universidad Católica del Ecuador, Quito, Ecuador; Departamento de Especialidades Médicas y Salud Pública, Universidad Rey Juan Carlos, Madrid, España; Programa María Zambrano, Universidad Rey Juan Carlos, Unión Europea-España. enriquegea@yahoo.es Pontificia Universidad Católica del Ecuador Pontificia Universidad Católica del Ecuador Quito Ecuador

**Keywords:** Occupational stress, burnout, medical staff *(source: MeSH, NLM)*, Estrés ocupacional, burnout, cuerpo médico *(fuente: DeCS, BIREME)*

## Abstract

**Objectives:**

To analyze the prevalence of Burnout Syndrome and identify sociodemographic and extra-occupational factors that influenced the development of this syndrome in medical personnel in Latin American countries during 2009-2020.

**Methods:**

Bibliographic research and a descriptive study approach included 35 articles that consider the Maslach Burnout Index to diagnose BOS. The relationship between the sample size, the prevalence and the corresponding subscales was carried out by means of a correlation analysis.

**Results:**

The presence of both sexes was balanced in most of the studies. Twenty-five studies addressed the relationship of sociodemographic and extra-occupational factors and their relationship with the development of BOS, while 14 of them identified one or more of these factors that were found to be influential or not at the time of diagnosis of the syndrome. The prevalence of BOS ranged from 2.4-83.3 %. There was no correlation between sample size and BOS (p=0.5993); identifying a positive correlation in the prevalence of the three subscales of the MBI, with values of r2 =0.72 between AE and DP (p<0.001); 0.39 for AE and RP (p=0.029), and 0.53 for DP and RP (p=0.002).

**Conclusions:**

Sociodemographic and extra-occupational factors are considered significant in predicting and diagnosing burnout. It is estimated that the factors studied should be linked to the diagnosis of BOS in early stages. Nevertheless, despite not being able to infer causality with these findings, they prove to be a useful basis for research and medical diagnosis in Latin America.

## RESUMEN

**Objetivos:**

Analizar la prevalencia e identificar factores sociodemográficos y extralaborales que influyeron en el desarrollo del síndrome de Burnout en personal médico en países de América Latina durante 2009-2020.

**Métodos:**

Investigación bibliográfica y estudio con enfoque descriptivo que incluyó 35 artículos que consideraban el índice de agotamiento de Maslach para diagnosticar SBO. La relación entre el tamaño muestral, la prevalencia de SBO y las subescalas correspondientes se llevó a cabo mediante un análisis de correlación.

**Resultados:**

La presencia de ambos sexos fue equilibrada en la mayor parte de los estudios. En 25 de ellos se abordó la relación de factores sociodemográficos y extra-laborales con el desarrollo del SBO, mientras que en 14 se identificaron uno o más de estos factores que resultaron ser influyentes o no al momento del diagnóstico del síndrome. Se obtuvo una prevalencia de SBO de entre 2,4 y 83,3 °%. No existió correlación entre el tamaño muestral y SBO (p=0,5993), y se identificó una correlación positiva en la prevalencia de las tres subescalas del MBI, con valores de r2 = 0,72 entre AE y DP (p<0,001); 0,39 para AE y RP (p=0,029), y 0,53 para DP y RP (p=0,002).

**Conclusiones:**

Los factores sociodemográficos y extralaborales se consideran significativos para predecir y diagnosticar el agotamiento. Se estima que se deberían vincular los factores estudiados con el diagnóstico del SBO en etapas tempranas. No obstante, a pesar de no poder inferir causalidad con estos hallazgos, resultan ser una base útil para la investigación y el diagnóstico médico en América Latina.

Burnout Syndrome (BOS) is defined as the process of responding to an excessive work overload that results in mental and psychological exhaustion of the worker 1. Due to the increased prevalence and health consequences of this syndrome, for those who suffer from it, BOS is recognized as the main mental health problem, derived from poor work environment management. Additionally, the impact on people's health, BOS has a considerable economic impact. According to the estimation of the World Economic Forum, the annual global expenditures associated with this syndrome are estimated to amount to 322 billion euros 2.

The main manifestations of BOS in affected individuals are the deterioration of interpersonal relationships, poor work performance and lack of commitment in their work area. All the above results in the wear-and-tear of the professional's mental health and can lead to psycho-somatic manifestations 3. Although BOS can impact any individual who performs medical assistance tasks, healthcare personnel are among the most affected 4. This is explained by the constant interaction with people with different ailments, the high number of hours in care work and the high responsibility of their work.

The increase in studies finding significant prevalence in physicians, residents and nursing staff in Latin American countries indicates that this is an issue that requires attention and control 5. Prevalence levels in countries within this region vary considerably, with reported values ranging from 7.7 % to 79.7 %, depending on the country and year 6. However, systematic, and standardized studies of the prevalence of BOS in this region, and the development of psychosocial factors in an individual, are still scarce 7. Unlike countries such as Spain, Germany and the United States in which strategies have been already designed and adopted to face this condition, in Latin American countries the responsibility to investigate and implement diagnostic, coping and prevention strategies should be a priority.

There are different scales or measurement systems to detect professional burnout. The Maslach Burnout Inventory (MBI) 1 is the most widespread, with 88 % of research publications on BOS using it as a source to identify and quantify the degree of professional burnout 8. The MBI scale includes 22 questions aimed to establish the individual's work situation within the job organization. The questions are grouped into the three characteristic dimensions of this syndrome: emotional exhaustion (EE), depersonalization (DP) and personal accomplishment (PA) 1. The wide use of this scale has allowed to understand the most common profiles of BOS, which are very useful when designing interventions for people with BOS.

Psychosocial factors include interactions of the individual at work, the work environment, and organizational conditions, in addition to their capabilities as a worker, their aspirations, their culture and their personal situation outside work. All these factors together can impact in health, physical performance, and job satisfaction 9. It is interesting that, both the MBI and other less widespread methods of assessment and diagnosis of BOS focus their research primarily on psychosocial factors associated with the work environment 1. This is explained by the fact that this syndrome originates in response to the social and organizational environment at work. As a result, within the studies aimed to determine the prevalence and psychosocial factors that influence the development of this syndrome, there is a tendency to underreport demographic factors. On the other hand, the contradictory results reported suggest that these factors may have different levels of influence in different countries.

Given the growing interest in gaining awareness in the detection and diagnosis of BOS in health care workers, the present study will focus on identifying the demographic and psychosocial factors that influenced the development of BOS in medical staff in Latin American countries from 2009 to 2020. As a result, it may contribute to improve the identification those at risk of developing this type of disorder which would allow earlier intervention in the course of the disease. Early detection of people with BOS can have a significant impact on reducing damage to the individual and the organization. It can also significantly shorten the recovery time of those treated, all of which reduces the economic cost of the disease.

The aim of this study is analyze the prevalence of BOS and the influence of extra-occupational factors on the incidence of this syndrome in Latin American physicians in the years 2009-2020.

## METHODS

### Research strategy

The literature search was performed in the databases Redalyc, Scielo, ScienceDirect and PubMed/Medline. The search included articles published in English, Spanish, and Portuguese in the years 2009 to 2020, using as keywords the term "Maslach Burnout Inventory" AND "physician" OR "physician".

### Study selection

This review was structured under the guidelines of the PRISMA Checklist from the TRANSPARENT REPORTING of SYSTEMATIC REVIEWS and META-ANALYSES. The articles identified were selected by inspection from title and abstract. Articles included in this study were carried out in Ecuador, Colombia, Suriname, Guyana, Chile, Argentina, Peru, Brazil, Uruguay, Paraguay, Venezuela, and Costa Rica, in which primary data were obtained on the results of the application of the MBI scale in physicians.

All studies, conducted in public and private institutions, included 20 or more physicians surveyed. Selected studies included those that used practitioner-applied or self-administered surveys. Duplicate studies were excluded because they were identified in different databases and published in different languages.

### Patient and public involvement

No patient was involved in the conduct of the study.

### Data extraction

Data extraction was performed by inspection of the full text. Each of the selected articles, the following data were extracted: number of people in the study, average age, working time, gender, prevalence of BOS and the three subscales of the MBI.

The prevalence of BOS was taken according to the diagnosis made by the authors of each research. For some authors, the presence of high scores in the EE was already enough to diagnose BOS. On the other hand, some authors considered the presence of BOS when there were significant scores for EE and DP, while other diagnosed BOS only when significant scores were obtained in the three subscales.

The extra-occupational sociodemographic factors analyzed were also recorded and classified as influential or not in the development of BOS.

### Statistical analysis

The data obtained were processed using the SPSS program (v.25.0) to conduct statistical analysis. The characterization of the different variables was performed by calculating the median and coefficient of variation, and the frequency, expressed as percentage values.

A correlation analysis was used to analyze the relationship between the prevalence of sample size and BOS, as well as the prevalence between the MBI scales (p<0.05 statistically significant).

## RESULTS

**Figure 1:**
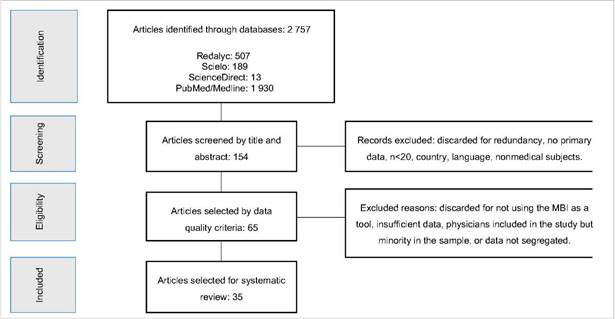
Flow diagram used in the identification, selection, eligibility and inclusion of articles. Diagram adapted from the PRISMA workflow^10^

The literature review identified a total of 2 757 articles published between 2009 and 2020 in which the search criteria "Maslach Burnout Inventory" appeared together with the words "physician" or "physician" (Figure 1). As a result of the title and abstract reading, 2 603 articles were excluded leaving a total of 154 studies in analysis. One of the main reasons of removing articles at this stage was the duplication in the results from different databases. We also removed research published in other languages but English, Spanish and Portuguese, studies with fewer than 20 subjects or in which the study population did not include graduate physicians.

Of the 154 articles selected, those that did not use the MBI as a tool to diagnose BOS or used an adaptation of this tool that has not been validated were excluded. We also excluded papers that included all the personnel of the care centers and the data referring to medical professionals could not be extracted independently from the other groups studied.

Finally, a total of 65 articles were selected for eligibility (Figure 1). Of these, we found that 35 studies evaluated BOS in physicians alone or together with other health care professionals (nurses and/or physical therapists and/or ancillary staff), however it allowed data to be extracted from the evaluation of physicians independently. From the inspection of the full texts, 35 studies were selected to extract data on the prevalence of BOS and its relationship with extra-occupational sociodemographic factors.

Table 1 shows the results of the overall sample and the total number of selected articles published between 2009 and 2020. The years 2016 and 2018 stand out with 7 and 6 articles selected. The final selection does not present any research published in 2010.

**Table 1:** Description of the studies included in the analysis

Country	Study	N	Age (Average)	Sex (%)		Prevalence (%)	BOS Subscales MBI (%)			Demographic factors/ diagnosis
				Female	Male		EA	DP	PA	
Argentina	(Burgos et al., 2018) 11	25	ND	42.1	57.9	26.1	56.5	52.2	69.6	Yes (yes)
	(Raúl et al., 2019) 12	213	45	54.5	45.5	15.9	64.8	39.9	23.5	Yes (yes)
	(Galv et al., 2012) 13	60	42	57.0	43.0	41	25	6	19	Yes (yes)
	(Waldman et al., 2009) 14	106	29	31.3	68.7	80.2	71.7	67.9	1.4	Yes (yes)
Brasil	(Pasqualucci et al., 2019) 15	429	28	ND	ND	63	63	63.5	49.2	No
	(Carneiro Monteiro et al., 2020) 16	66	28	47	53	83.3	47	62.1	69.7	No
	(de Novais et al., 2016) 17	43	45	4.7	95.3	46.5	ND	ND	ND	No
	(Lima et al., 2018) 18	134	ND	25.2	47.8	10.4	6	44.8	28.4	Yes (yes)
	(Garcia et al., 2014) 19	70	36	21	79	20	44	24.2	17	Yes (n o)
	(De Andrade et al., 2016) 20	32	ND	78	28	53	17	19	31	Yes (yes)
	(Barbosa et al., 2012) 21	67	43	55.2	44.8	70.1	41.8	37.3	58.2	Yes (ND)
	(Cubero et al., 2016) 22	54	28	46.3	53.7	76	49	64.7	ND	Yes (ND)
	(Paiva et al., 2018) 23	227	34	ND	ND	58.1	41.9	37.6	50.9	Yes (n o)
	(Ren et al., 2013) 24	297	34	28.1	71.9	63.4	47.6	24.7	28.4	Yes (yes)
	(Freire et al., 2016) 25	198	49	31.5	68.5	48.7	26.9	41.3	37.2	Yes (yes)
	(Da Cruz Gouveia et al., 2017) 26	129	ND	51.9	48.1	27.9	59.7	31.8	94.6	Yes (n o)
	(Da Silva et al., 2017) 27	78	ND	87.2	12.8	23.1	42.3	38.5	6.4	Yes (ND)
	(Barbosa et al., 2017) 28	43	49	48.8	51.2	67.4	25.6	44.2	51.2	Yes (ND)
	(Martins et al., 2011) 29	74	27	81	19	66	ND	ND	ND	No
	(Tironi et al., 2016) (RS 30	180	39	54.4	45.6	61.7	50.6	26.1	15.0	No
	(Magalhães et al., 2015) 31	134	40	34.3	65.6	10.4	23.1	28.3	47.7	Yes (yes)
	(Sousa et al., 2018) 32	48	42	42.3	57.7	2.4	24.4	29.3	34.1	No
	(Zétola et al., 2019) 33	74	45	47.3	52.7	ND	26.2	28.6	4.8	Yes (n o)
Costa Rica	(Syndrome & Residents, 2009) 34	45	28	43.6	56.4	72	21.8	10.7	13.6	Yes (n o)
Chile	(Astudillo M. et al., 2018) 35	94	33	31	69	64.4	76	62	55	Yes (yes)
Colombia	(Hernández et al., 2021) 36	117	44	57.3	42.7	ND	3.4	4.3	4.3	No
	(Dávila & Nevado, 2016) 37	73	25	49	51	28	ND	ND	ND	No
Ecuador	(Ramírez et al., 2018) 38	2404	40	68.4	31.6	2.6	17.2	13.5	18.2	Yes (yes)
	(Ruisoto et al., 2021) 39	608	40	68	32	12	19	1	7.5	No
Perú	(Beas et al., 2014) 40	2228	45	23.8	76.2	2.8	10.6	12.8	19.4	Yes (n o)
	(Huarcaya-Victoria & Calle-Gonzáles, 2020) 41	145	32	ND	ND	9.7	40	33.8	15.9	Yes (yes)
	(Gastelo-salazar et al., 2020) 42	138	ND	49.3	50.7	28	ND	ND	ND	Yes (n o)
Uruguay	(Burghi et al., 2014) 43	82	ND	ND	ND	51	ND	ND	ND	Yes (yes)
Venezuela	(Arayago et al., 2016) 44	64	29	62.5	37.5	64.1	34.4	51.6	81.2	No
	(Orozco et al., 2012) 45	88	ND	61.4	38.6	56.7	63	49	36	Yes (yes)

N: number of people included in the study.

ND: not determined.

EA: emotional exhaustion.

DP: depersonalization.

**Figure 2:**
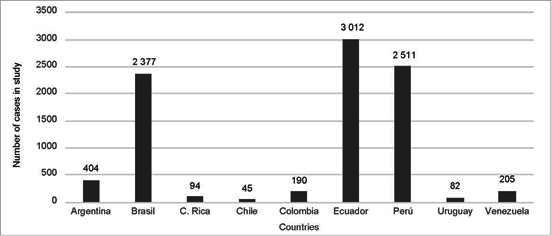
Number of individuals included in studies published between 2009 and 2020

A highly heterogeneous sample was obtained in the number of investigations selected by country (Table 1). Of the total number of articles included in the review, 4 corresponded to research carried out in hospital institutions in Argentina, 1 in Costa Rica, 1 in Chile, 2 in Colombia, 2 in Ecuador, 3 in Peru, 1 in Uruguay, 2 in Venezuela, with Brazil standing out with 19. However, the sample size does not correspond to the number of articles per country (Table 1). Countries such as Ecuador and Peru, with 2 and 3 articles selected respectively, present larger sample sizes (11 = 3012 and 1 = 2511, respectively) than Brazil (1=2377). The sample sizes of Ecuador and Peru respond to the fact that for both countries articles were found that conducted studies at the national level. The remain countries, the sample size is more closely related to the number of articles included: Argentina with a sample size of 404 individuals, Venezuela with 205, Colombia with 190, Costa Rica with 94, Uruguay with 82, and Chile with 45 (Figure 2).

The average age of the individuals surveyed in the selected studies ranged from 25 to 49 years (Table 1), with the median of 39 and a coefficient of variation (CV) of 20.3 %. The representation of each gender is balanced in most studies with 15 (46.8 %) studies containing 40-60 %o female sex; 6 (18.7 %>) with 60-80 %> and 8 (25 %>) with 20-40 %. Some studies with an asymmetrical representation of both genders stand out. Such is the case of the study by De Novais et al. 2016, with 4.7 % of females in a sample of 43 cases, and the investigations by Da Silva et al. 2017 and Martins et al. 2011, with 87 % and 81 % of females in a total of 78 and 74 individuals included in the study, respectively (Table 1).

**Figure 3:**
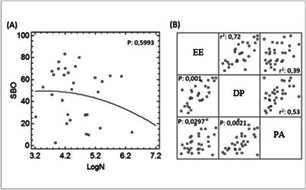
Correlation analysis. (A) Sample size (N) and prevalence of BOS found for each study. (B) Relationship in the prevalence of the MBI subscales. EE, emotional exhaustion. DP, depersonalization. PA, personal development

The prevalence of BOS varied considerably, with interval values from 2.4 % to 83.3 % (Table 1). No correlation was found between sample size (N) and BOS, despite transforming the data to obtain a normal distribution (p=0.993, Figure 3A). Similarly, significant heterogeneity is observed in the prevalence of impairment in the subscales of the MBI with frequencies between 3.4 % and 79 % for EE; between 1 % and 68 % for DP, and between 1.4 % and 94.6 % for PA (Table 1). A positive correlation was found in the prevalence of the three subscales of the MBI with r2 values =0.72 between EE and DP (p<0.001); 0.39 for EE and RP (p = 0.029), and 0.53 for DP and RP (p=0.0021) (Figure 3B).

Twenty-five studies (71.4 %) address the relationship of extra-occupational sociodemographic factors in relation to the development of BOS (Table 1). In 14 (56 %) of them, one or more extra-occupational factors are found to be prevalent, either as stressors or protectors. The extra-occupational factors studied include age, sex, marital status, children, the practice of sports and physical activities, living with family, professing a religion, hours of relaxation practicing a hobby or meeting with friends, place of residence (metropolitan area or not) and home ownership or not (Table 2).

**Table 2:** Sociodemographic factors outside work studied

Factor	Number of articles Total (%)^a^	Number of articles Factors (%)^b^
Age	15 (42)	4 (27)
Sex	20 (57)	5 (25)
Marital status	16 (45.7)	6 (37.5)
Sons	11 (31.4)	1 (9)
Physical activity	7 (20)	2 (28.6)
Coexistence	9 (25.7)	3 (33)
Religion	3 (8.5)	0
Hours relaxaction/hobby	4 (11. 4)	1 (25)
Residence	6 (17.1)	2 (33)
Property living place	3 (8.5)	0

^a^Referred to the percentage of the total number of studies included, N=35 (Table 1).

^b^Referred to the percentage of the total number of studies that address extra-occupational sociodemographic factors among the study variables, N=25 (Table 1).

## DISCUSSION

BOS is a syndrome lead by poor management of workload and work environment 1. For healthcare personnel, the emotional burden derived from the constant interaction with people suffering from illnesses, sometimes have low chances to be solved. All these factors make healthcare personnel more prone to suffer this syndrome 46.

Previous studies have shown differences of features in the BOS development between physicians and nurses in the same hospital institution 47. This shows that the healthcare workforce professionals with different job profiles and training may perceive and respond differently to the same stressors. Hence the importance of conducting studies focused on each of the professional profiles will help to establish more effective strategies to prevent and control the occurrence of this syndrome.

Within the healthcare workforce, physicians have a high burden of patient interaction and direct responsibility for outcomes of care. They also have bigger risks to develop BOS within hospital institutions. In fact, it is estimated that one in three physicians experience burnout at any given time 46.

In general physicians play a central role in the quality of healthcare services. A high prevalence of BOS in medical staff may not only interfere with their own well-being, but also with patient satisfaction and safety. Additionally, the complications of the medical staff's service results in significant economic impacts for the afflicted person and for the institution. As an example, the cost per year associated with BOS in physicians in the United States has been estimated at $5 billion 48.

Consequently, it is essential to focus the present work to identify the different sociodemographic and extra-occupational factors that contribute the development of BOS in medical personnel. When sociocultural differences are recognized in Latin American countries, many development programs at the regional level are aimed to create conditions more conducive to commercial exchange and, in general, to economic and social integration. Therefore, it is important to recognize the common points that affect the performance of different society sectors to contribute the development of programs to improve working conditions that have a regional scope. This is a particular interest at the present time when the COVID-19 pandemic is creating great pressure on all health systems and their professionals 49.

Considering the sample size of all the sources consulted, the present work summarizes the prevalence rates identified for a total of 8949 health professionals, specifically medical personnel from nine Latin American countries: Argentina, Brazil, Chile, Costa Rica, Colombia, Ecuador, Peru, Uruguay, and Venezuela (Table 1). In general, the results show a high prevalence of BOS in the period 2009-2020 and confirm the relevance of conducting studies aimed to determine factors that trigger the development of this syndrome in medical personnel.

Seven studies from Argentina (2 studies), Brazil (3), Colombia (1) and Peru (1), reported a prevalence of BOS in medical staff between 25-50 %; similar to figures reported in another regions 50-52. However, a total of sixteen studies among publications from different countries, Argentina (1), Brazil (10), Chile (1), Costa Rica (1), Uruguay (1) and Venezuela (2), found a prevalence of more than 50 % in medical personnel. Only ten studies reported less than 25 % prevalence (Table 1).

The differences found in the different institutions and countries of development of the prevalence of BOS in different studies should be interpreted carefully since they may not reflect real differences in the frequency of occurrence of this disease. The efforts to obtain comparable results that would reflect the prevalence of BOS in relation to sociodemographic factors in different countries and places in Latin America. The methodology used established the inclusion of only those studies that used the MBI scale to diagnose BOS. However, even in the application of this scale, there are important differences.

The 10 studies that found a prevalence of BOS of less than 25 %, 8 used as criteria for the diagnosis of BOS that the three subscales of the MBI were altered simultaneously, i.e. EE, DP or PA. In contrast to this, the studies that found a higher prevalence of BBS took as a condition for diagnosis that the person presented only one of the three altered domains. The discordance in the criteria of the diagnosis of BOS has been found in other literature studies 53, and it is recognized as one of the main limitations that exist to define a prevalence rate at regional levels 46.

Another source of divergence in the included studies is sample size. A smaller sample size may result in lower sensitivity to detect the prevalence of BOS, as well as the impact of sociodemographic factors. However, the correlation analysis performed evidenced that there is no bias in the prevalence of BOS in relation to the number of people included in each study (Figure 3A).

On the other hand, the correlation obtained between the different components of the MBI scale (Figure 3B) implies that there is a higher chance that individuals in whom only one of the altered subscales was identified were individuals in early stages of BOS development, still subclinical, and may make detection through the interview difficult. For this reason, both the studies that made the diagnosis based on the alteration of one of the factors and those that made the diagnosis only when the three subscales were altered were considered.

To contribute the development of effective programs for the prevention of BOS it is required an intervention that encompasses the personal response, the organizational context, and the interaction between the organization and the individual. Hence, it is necessary to understand not only the factors inherent to the organization and work environment, but also those associated with culture, interpersonal relationships, family and home 46.

The possible influence of extra-work sociodemographic factors such as gender and family on the development of BOS was recognized from the initial work on the implementation of the MBI scale 54. In fact, sex, age, marital status and number of children were found to be among the most frequently analyzed factors, with values of 57 %, 42 %, 45.7 % and 31.4 % respectively, out of 25 papers that analyzed extra-occupational factors in our analysis.

It has been found that men and women experience burnout differently. Generally speaking, women tend to experience greater EE, while men are more likely to experience DP 55. Age may influence the occurrence of burnout and maturity carries out better available strategies of emotional self-control, as a result, the person is less impressionable 56. Marital status and having children can also influence, because professionals in this condition can find goals beyond work, allowing them to find strength in other areas of life and providing them motivation to cope their work 57. However, when family life and work interfere with each other, BOS can occur 58.

The particular interest that other factors included in a more limited number of studies such as physical activity (20 %), hours of relaxation (11.4 %), living together (25.7 %), and place of residence (17.1 %), showed influence on the development of BOS with frequencies between 25 % and 33 %; similar to those of the commonly analyzed factors. Physical activity has been identified as one of the best ways to cope BOS because it promotes physical benefits, controls stress, and reduces anxiety and depression levels 59. Likewise, quality time off, time to share with friends as well as a well-balanced family relationship can contribute to offset the stress derived from work relationships.

The influence of the place of residence has been analyzed from several angles, including elements related to the proximity to the workplace and the quality of life it offers, i.e. satisfaction with basic services. Elements such as close relationships with family may favor greater emotional stability, considering this interaction as an element that contributes to reduce over-involvement in work. It has also been observed that the greater the proximity to the workplace, the lower the burnout index. Following this last observation, results of a study proved that 83.5 % of the individuals surveyed stated that they would not take a job at a distance of 80 km from their place of residence and only 16.5 % said that they would take it 60.

A remarkably high prevalence of BOS is identified in the medical personnel of several countries of Latin America in the last decade, but sociodemographic factors outside of work are still underrepresented in the prevalence studies of BOS. There is no correspondence between the extra-work factors included more frequently in the studies (sex, marital status, and age), with the factors found with the greatest incidence in the development of BOS (marital status, physical activity, coexistence, and location of residence).

The results obtained evidence the need to assess the risk of BOS and explore possible influence on the development of this syndrome in medical staff including a broad spectrum of sociodemographic and extra-occupational factors. Studies on burnout in healthcare professionals conducted at the regional level may achieve standardization of the extra-occupational factors analyzed and greater information regarding their influence 51. All this could contribute to achieve more effective BOS detection and treatment programs by being based on well-established evidence and not only on the empirical context that is currently accumulating ♦

## References

[1] Maslach C, Jackson SE (1981). The measurement of experienced burnout. J Organ Behav.

[2] Bruce J (2019). The overlooked consequences of today's burnout.

[3] Schaufeli WB Leiter MP, Maslach C (2009). Burnout: 35 years of research and practice. Career Dev Int.

[4] Lloyd C, King R, Chenoweth L (2002). Social work, stress and burnout: A review. J Ment Health.

[5] Bitran M, González M, Nitsche P, Denisse Z, Riquelme A (2017). Concern for residents' wellbeing, an issue discussed at the Latin American Conference on Resident Education (LACRE) 2017. Rev Med Chil.

[6] García JE, García DA (2010). Prevalencia del síndrome de agotamiento profesional en médicos familiares mexicanos: análisis de factores de riesgo. Rev Colomb Psiquiatr.

[7] García-Arroyo J, Osca Segovia A (2018). Effect sizes and cut-off points: a meta-analytical review of burnout in Latin American countries. Psychol Health Med.

[8] Boudreau RA, Boudreau WF, Mauthe-Kaddoura AJ (2015). From 57 for 57: A bibliography of burnout citations.

[9] Toro Álvarez F, Londoño Londoño ME, Sanín Posada A, Valencia Jáuregui M (2010). Modelo analítico de factores psicosociales en contextos laborales. Rev Interam Psicol Ocup.

[10] Page MJ, Moher D, Bossuyt PM, Boutron I, Hoffmann TC, Mulrow CD (2021). PRISMA 2020 explanation and elaboration: Updated guidance and exemplars for reporting systematic reviews. BMJ.

[11] Burgos LM, Battioni L, Costabel JP, Alves de Lima A (2018). Evaluation of burnout syndrome in medical residents following a "rest after shift" intervention. Rev Argent Cardiol.

[12] Zuin DR, Peñalver F, Zuin MP (2020). Síndrome de burnout o de agotamiento profesional en la Neurología argentina. Resultados de una encuesta nacional. Neurol argent.

[13] Galván ME, Vassallo JC, Rodríguez SP, Otero P, Montonati MM, Cardigni G (2012). Síndrome de desgaste profesional (burnout) en médicos de unidades de cuidados intensivos pediátricos en la Argentina. Arch Argent Pediatr.

[14] Waldman SV, Lopez Diez JC, Arazi HC, Linetzky B, Guinjoan S, Gran-celli H (2009). Burnout, perceived stress, and depression among cardiology residents in Argentina. Acad Psychiatry.

[15] Pasqualucci PL, Mendes Damaso LL, Danila AH, Fatori D, Neto FL, Kalika Koch VH (2019). Prevalence and correlates of depression, anxiety, and stress in medical residents of a Brazilian academic health system. BMC Med Educ.

[16] Carneiro Monteiro GM, Passos IC, Baeza FLC, Hauck S (2020). Burnout in psychiatry residents: The role of relations with peers, preceptors, and the institution. Braz J Psychiatry.

[17] De Novais RN, Rocha LM, Eloi RJ, Dos Santos LM, Moura Rezende Ribeiro MV, DA Silva Ramos FW (2016). Burnout syndrome prevalence of on-call surgeons in a trauma reference hospital and its correlation with weekly workload: Cross-sectional study. Rev Col Bras Cir.

[18] Lima CRC, Sepúlveda JLM, Lopes PHTNP, Fajardo HSR, de Sousa MM, Ferreira MC (2018). Prevalence of burnout syndrome among military physicians at a public hospital in Rio de Janeiro, Brazil. Rev Bras Med Trab.

[19] Garcia TT, Garcia PCR, Molon ME, Piva JP, Tasker RC, Branco RG (2014). Prevalence of burnout in pediatric intensivists: An observational comparison with general pediatricians. Pediatr Crit Care Med.

[20] De Andrade APM, Amaro E, Farhat SCL, Schvartsman C (2016). Higher burnout scores in paediatric residents are associated with increased brain activity during attentional functional magnetic resonance imaging task. Acta Paediatr.

[21] Barbosa FT, Leão BA, Tavares GMS, Peixoto dos Santos JGR (2012). Burnout syndrome and weekly workload of on-call physicians: cross-sectional study. Sao Paulo Med J.

[22] Cubero DIG, Fumis RRL, de Sá TH, Dettino A, Costa FO (2016). Adam Van Eyll BMRH, et al. Burnout in medical oncology fellows: a prospective multicenter cohort study in Brazilian institutions. J Cancer Educ.

[23] Paiva CE, Martins BP, Paiva BSR (2018). Doctor, are you healthy? A cross-sectional investigation of oncologist burnout, depression, and anxiety and an investigation of their associated factors. BMC Cancer.

[24] Tironi MOS, Sobrinho CLN, Barros DS, Borges Reis EJF, Marques ES, Almeida A (2010). Professional burnout syndrome among intensive care Physicians in Salvador, Brazil. Rev Assoc Med Bras.

[25] Freire PL, Trentin JP, De Avila Quevedo LA (2016). Trends in burnout syndrome and emotional factors: An assessment of anesthesiologists in Southern Brazil, 2012. Psychol Health Med.

[26] Da Cruz Gouveia PA, Neta MHCR, De Moura Aschoff CA, Pires Gomez D, Fonseca da Silva NA, Firmino Cavalcanti HA (1992). Factors associated with burnout syndrome in medical residents of a university hospital. Rev Assoc Med Bras.

[27] Da Silva DKC, De Jesus Torres Pacheco M, Marques HS, Castelo Branco RC, Neto da Silva MAC (2017). Brandão Nascimento MDS. Burnout no trabalho de médicos pediatras. Rev Bras Med Trab.

[28] Barbosa FT, Eloi RJ, dos Santos LM, Leão BA, Camelo de Lima FJ, de Sousa-Rodrigues CF (2017). Correlation between weekly working time and burnout syndrome among anesthesiologists of Maceió-AL. Braz J Anesthesiol.

[29] Martins AE, Davenport MC, Del Valle MPLP, Di Lalla S, Domínguez P, Ormando L (2011). Impact of a brief intervention on the burnout levels of pediatric residents. J Pediatr.

[30] Tironi MOS, Teles JMM, De Souza Barros D, Bôas Vieira DFV, da Silva Filho CM, Martins DF (2016). Prevalence of burnout syndrome in intensivist doctors in five Brazilian capitals. Rev Bras Ter Intensiva.

[31] Magalhães E, Oliveira ÁCM de S, Govêia CS, Araújo Ladeira LC, Queiroz DM, Vieira CV (2015). Prevalência de síndrome de burnout entre os anestesiologistas do Distrito Federal. Braz J Anesthesiol.

[32] Govêia CS, Mendes da Cruz TT, Borges de Miranda D, Nunes Guimarães GM, Araújo Ladeira LC, Sampaio Tolentino FD (2018). Associacão entre síndrome de burnout e ansiedade em residentes e anestesiologistas do Distrito Federal. Braz J Anesthesiol.

[33] étola VF, Pavanelli GM, Pereira GU, Branco Germiniani FMB, Lange MC (2019). Burnout syndrome: Are stroke neurologists at a higher risk?. Arq Neuropsiquiatr.

[34] Millán-González R, Mesén-Fainardi A (2009). Prevalencia del síndrome de desgaste en médicos residentes costarricenses. Acta Méd Costarric.

[35] Astudillo P, Losada H, Schneeberger P, Coronado F, Curitol S (2018). Prevalencia de síndrome de burnout en un centro de cirugía académico-asistencial público en Chile. Rev Chil Cir.

[36] Hernández SM, Patiño C, Carreño M, Aranzazu-Moya GC, Rodríguez MJ (2022). Factores asociados con el agotamiento psicológico en odontólogos especialistas colombianos. Rev Colomb Psiquiatr.

[37] Dávila FA, Nevado N (2016). Validation of the burnout screening inventory in health area trainees. Educ Méd.

[38] Ramírez MR, Otero P, Blanco V, Ontaneda MP, Díaz O, Vázquez FL (2018). Prevalence and correlates of burnout in health professionals in Ecuador. Compr Psychiatry.

[39] Ruisoto P, Ramírez MR, García PA, Paladines-Costa B, Vaca SL, Clemente-Suárez VJ (2021). Social support mediates the effect of burnout on health in health care professionals. Front Psychol.

[40] Beas R, Anduaga-Beramendi A, Maticorena-Quevedo J, Mayta-Tristán P (2017). Associated factors with burnout syndrome in physicians and nurses from Peru, 2014. Rev Fac Cienc Méd Córdoba.

[41] Huarcaya-Victoria J, Calle-Gonzáles R (2021). Influencia del síndrome de burnout y características sociodemográficas en los niveles de depresión de médicos residentes de un hospital general. Educ Méd.

[42] Gastelo-Salazar KY, Rojas-Ramos AP, Díaz-Vélez C, Maldonado Gómez W (2020). Hospital educational climate and burnout syndrome in foundation years. Educ Méd.

[43] Burghi G, Lambert J, Chaize M, Goinheix K, Quiroga C, Fariña G (2014). Prevalence, risk factors and consequences of severe burnout syndro-me in ICU. Intensive Care Med.

[44] Arayago R, González A, Limongi M, Harold G (2016). Síndrome de burnout en residentes y especialistas de anestesiología. Salus.

[45] Orozco G, Chacín MD, González E, Coruj M (2012). Estudio comparativo del desgaste profesional en residentes de Postgrado Clínico de la Universidad de Carabobo. Venezuela. Informe Médico.

[46] De Hert S (2020). Burnout in healthcare workers: Prevalence, impact and preventative strategies. Local Reg Anesth.

[47] Miranda Alvares ME, Fonseca Thomaz EBA, Lamy ZC, de Abreu Haickel Nina RV, Lopez Pereira MU, Santos Garcia JB (2020). Burnout syndrome among healthcare professionals in intensive care units: A cross-sectional population-based study. Rev Bras Ter Intensiva.

[48] Han S, Shanafelt TD, Sinsky CA, Awad KM, Dyrbye LN, Fiscus LC (2019). Estimating the attributable cost of physician burnout in the United States. Ann Intern Med.

[49] Magnavita N, Chirico F, Garbarino S, Luigi Bragazzi N, Santacroce E, Zaffina S (2021). SARS/MERS/SARS-CoV-2 outbreaks and burnout syndrome among healthcare workers. An umbrella systematic review. Int J Environ Res Public Health.

[50] Low ZX, Yeo KA, Sharma VK, Leung GK, McIntyre RS, Guerreo A (2019). Prevalence of burnout in medical and surgical residents: a meta-analysis. Int J Environ Res Public Health.

[51] Rotenstein LS, Torre M, Ramos MA, Rosales RC, Guille C, Sen S (2018). Prevalence of burnout among physicians: a systematic review. JAMA.

[52] Yates SW (2020). Physician stress and burnout. Am J Med.

[53] Cardona CA, Alberto C, Castrillón C, Jaime J, Arango CA, Rodríguez D (2011). Prevalencia y factores psicosociales asociados al síndrome de Burnout en médicos que laboran en instituciones de las ciudades de Manizales y La Virginia (Colombia) 2011. Arch Med.

[54] Maslach C, Jackson SE (1985). The role of sex and family variables in burnout. Sex Roles.

[55] Purvanova RK, Muros JP (2010). Gender differences in burnout: a meta-analysis. J Vocat Behav.

[56] Aguirre Roldán AM, Quijano Barriga AM (2015). Burnout syndrome, family and work related variables on general practitioners in Bogota. A Strategy of work quality. Rev Colomb Psiquiatr.

[57] Jones ML (1993). Role conflict: cause of burnout or energizer?. Soc Work.

[58] Allen TD, Herst DEL, Bruck CS, Sutton M (2000). Consequences associated with work-to-family conflict: a review and agenda for future research. J Occ Health Psychol.

[59] Josefsson T, Lindwall M, Archer T (2014). Physical exercise intervention in depressive disorders: meta-analysis and systematic review. Scand J Med Sci Sports.

[60] Guercovich A, Piazzoni G, Guercovich J, Piazzoni L, Peñaloza J, Beguelin Z (2017). Prevalencia del síndrome de burnout en oncólogos clínicos asistentes a la XXVI reunión de trabajos y actualización post Chicago de la Asociación Argentina de Oncología Clínica, 2016. Oncol Clín.

